# The Association Between Conducting a Multidisciplinary Team Conference and Short- and Long-Term Outcomes After Colorectal Cancer Surgery: A National Register Study

**DOI:** 10.1245/s10434-025-18353-y

**Published:** 2025-09-30

**Authors:** Karoline Bendix Bräuner, Maliha Mashkoor, Mikail Gögenur, Viviane Lin, Carolin Oppermann, Ismail Gögenur

**Affiliations:** 1 Center for Surgical Science, Zealand University Hospital, Køge, Denmark; 2 The Faculty of Health Science, University of Copenhagen, Copenhagen N, Denmark

**Keywords:** Colorectal cancer, Machine learning, Multidisciplinary team, Cancer care

## Abstract

**Background:**

Elective treatment of colorectal cancer (CRC) is usually determined at a multidisciplinary team (MDT) conference. This practice has been registered in the Danish Colorectal Cancer Group database since 2010 and recommended in their guidelines since 2016. We aimed to investigate outcomes after MDT implementation.

**Methods:**

We converted four nationwide observational health databases into a Common Data Model. We compared patients with and without an MDT discussion before CRC surgery using propensity score matching at a ratio of 1:1, and matched based on conditions at all times before surgery, procedures and observations 5 years before surgery, and medications 30 days before surgery. Outcomes were complications, return to intended oncological therapy (RIOT), mortality, and disease-free survival (DFS).

**Results:**

We matched 3143 patients in each group who underwent elective surgery for CRC with curative intent between 2010 and 2019 and 995 patients from 2010 to 2019 eligible for adjuvant therapy. For RIOT at 90 days postoperatively, we calculated hazard ratios (HRs) of 1.04 (95% confidence interval [CI] 0.909–1.18) for the MDT group, 0.952 (95% CI 0.812–1.12) for 1-year mortality, and 0.951 (95% CI 0.868–1.04) for 5-year mortality. In the MDT group, the HR for DFS was 0.952 (95% CI 0.853–1.06), while the odds ratio (OR) for complications within 30 days of surgery was 0.982 (95% CI 0.676–1.43) and the OR for the proportion of patients receiving adjuvant therapy was 1.02 (95% CI 0.92–1.13).

**Conclusions:**

MDT discussions were not significantly associated with differences in short- and long-term outcomes.

**Supplementary Information:**

The online version contains supplementary material available at 10.1245/s10434-025-18353-y.

The cornerstone of curative treatment of colorectal cancer (CRC) is surgical resection, but a combination of surgery and other treatment modalities such as radiation therapy, chemotherapy, and immunotherapy have resulted in considerable improvements in disease-free survival (DFS) for an increasing number of subgroups of patients.^1,2^ To optimize cancer patient trajectories, surgical national and international societies have recommended that all patients with a malignant disease should be discussed in a multidisciplinary team (MDT) conference, and this recommendation is based on studies assessing the effects of MDT conferences on patient outcomes.^3–5^

MDT conferences can occur at multiple times in the cancer trajectory, including preoperatively, preoperatively after neoadjuvant therapy, postoperatively, or when metastasis is identified. Multidisciplinary discussion in a highly specialized tumor board is required, and during follow-up if there is suspicion of recurrence. In CRC care in Denmark, the preoperative MDT conference is prioritized since this is where decisions about surgery are generally made. During the MDT conference, the clinical team will assess the advancement of disease and data on the general health of the patient, and, using this knowledge, decisions will be made regarding surgical and oncological strategies. Since the Danish guidelines recommend that MDT discussions be conducted for all patients with newly diagnosed CRC, it is important to know how these discussions impact patient outcomes.

Reconvalescence after surgery may have both short- and long-term consequences; for instance, risk of chronically reduced functional capacity and postoperative complications,^1^ postoperative mortality, and delays in the start of adjuvant therapy in patients with higher-stage disease.^6^ The latter is known as return to intended oncological therapy (RIOT).^7,8^ It has been suggested that RIOT, defined as the number of patients receiving adjuvant chemotherapy and time to start of treatment, is associated with long-term survival after cancer surgery.

Although the implementation of recommended MDT conferences provides a platform for more personalization of care,^9,10^ only a few studies have assessed possible benefits for patients discussed in an MDT meeting versus patients who were not.^3^ This study aimed to assess long- and short-term clinical outcomes for patient trajectories in those with CRC who underwent MDT discussion compared with those who did not.

## Methods

### Study Design

We performed a retrospective cohort study in which patients who did and did not undergo an MDT conference were propensity score matched, and their outcomes were subsequently assessed. For this study, we linked four Danish observational health databases before matching the cohorts. We followed the Strengthening the Reporting of Observational Studies in Epidemiology (STROBE) guidelines for this study.^12^

### Registries

The Danish Colorectal Cancer Group (DCCG) database,^13,14^ The Danish National Patient Registry (DNPR),^15^ The Register of Laboratory Results for Research (RLRR),^16^ and the National Prescription Database (NPR)^17^ were included in this study to highlight multiple aspects of the patient phenotype and trajectory. All four of these registers are nationwide and include patients from all five Danish Regions. The DNPR, RLRR, and NPR are governed by the Danish Health Authorities and are populated through connection to the patients’ electronic health records, ensuring very high coverage, while the DCCG database is based on manual registration. Data granularity in the DCCG database has been shown to be very high and the register has a completeness of 95% (2001–2010) to 99% (2010 and onwards).^13,14^

Using nationwide databases allowed for the inclusion of all 13 Danish CRC centers in the analysis. Since 2014, the variable ‘MDT conference’ has been included in the DCCG database and has ‘yes’, ‘no’, and ‘unknown’ responses. We found that 30% of patients had unknown MDT status and were therefore excluded in order to ensure that ‘no MDT’ indeed meant that there was no MDT at all. No details on team members were included in the data but all centers were assumed to use at least a surgeon, an oncologist, a radiologist, and a pathologist, as recommended by Danish guidelines.

The DCCG register was established in 2001 and has since been updated several times, incorporating an increasing number of variables (currently exceeding 400 perioperative variables). Data were collected prospectively and were manually inputted by the responsible colorectal surgeons, although some variables were collected from other databases. Since commencement of the DCCG database in 2001 and until the last patient in 2019, 76,849 patients had been registered in the database,^18^ of whom 65,612 patients underwent surgery.

Established in 1977, the DNPR includes trajectory data on all patients admitted to a Danish hospital,^15^ mainly including procedures and diagnoses.

The RLRR and NPR databases are based on the surveillance of laboratory samples or purchased prescription medicine, respectively.^16,17^ These registers contain no diagnosis or procedure information. Nonetheless, in combination with the DCCG database and the DNPR, which provide an understanding of the patient’s phenotype, information from the RLRR and NPR can provide a further understanding of competing illnesses. As with the DNPR, information from these two databases is only included for patients who were also present in the DCCG database.

All four databases were transformed into an Observational Medical Outcomes Partnership Common Data Model (OMOP CDM).^19–21^

### Study Population

**Fig. 1:**
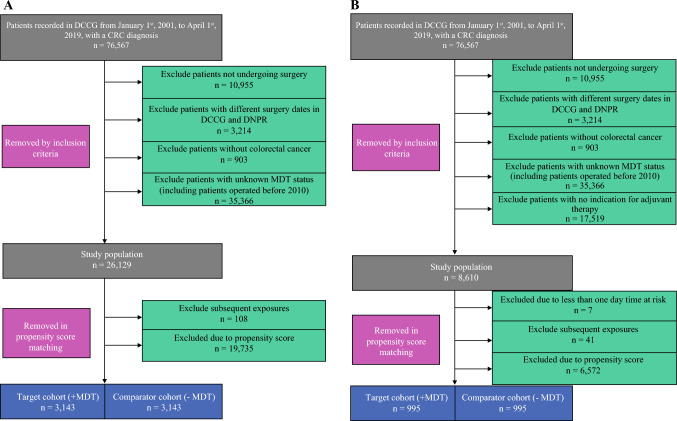
Patient inclusion before and after propensity score matching for (**a**) mortality, disease-free survival, and major surgical complications; and (**b**) return to intended oncological therapy. *DCCG* Danish Colorectal Cancer Group, *CRC* colorectal cancer, *DNPR* Danish National Patient Registry, *MDT* multidisciplinary team

Two study cohorts were designed. The first cohort assessed 1- and 5-year all-cause mortality, complications, and DFS outcomes in patients over 18 years of age who had a CRC diagnosis and underwent elective surgery with curative intent. Patients in the MDT group must have had an MDT event within 60 days before surgery, whereas patients in the comparator group did not have an MDT conference within 60 days before surgery. The second cohort included patients with a pathological Union for International Cancer Control (UICC) stage of III or higher, defined as a minimum of one of the following criteria: pT4, pN1, pN2, cM1, or pM1 with proficient mismatch repair (pMMR). For both groups, patients were included from 1 January 2014 until 31 December 2019. The patient inclusion flowcharts are visualized in Fig. 1.

### Outcomes

We investigated the association between discussing the treatment plan in the preoperative MDT conference and five specific outcomes. Outcomes were separated into short-term outcomes, including major surgical complications within 30 days of surgery and RIOT within 90 days of surgery, and long-term outcomes, including 1- and 5-year mortality as well as DFS within 5 years of the primary surgery. Statistically significant differences are marked with an asterisk (*).

#### Short-Term Outcomes

The first short-term outcome was the association between the preoperative MDT discussion and major surgical complications. Major surgical complications were defined as Clavien–Dindo grade 3b or higher within 30 days of surgery,^11^ and also included registration of reoperation and death. The risk time started from the date of surgery and ended 30 days after. If any complications meeting these criteria were registered, the outcome was positive regardless of any subsequent complications.

Second, for the patient group with UICC stage III or higher disease undergoing curative-intent surgery, we investigated the association between preoperative MDT discussion and RIOT after surgery, defined as the first record of cytostatic or radiation therapy in the DCCG database or the DNPR within 90 days of surgery. In a time-to-event context, RIOT as an outcome is defined as the time (in days) from surgery to the initiation of either preoperative or adjuvant therapy. In a binary context, RIOT as an outcome refers to the percentage of eligible patients who actually received adjuvant therapy. For this study, we assessed both the time-to-event and the percentage of eligible patients receiving adjuvant therapy. This time-at-risk was selected because the Danish national guideline for adjuvant therapy for CRC calls for the initiation of therapy, optimally within 4 weeks of surgery and generally not after 3 months of surgery. This would also avoid the inclusion of patients referred to palliative oncological therapy later in the trajectory, for instance due to metastatic recurrence of the disease. We censored all patients who did not complete the 90-day time at risk, therefore potential short-course deaths were not counted as not being referred.

#### Long-Term Outcomes

DFS was defined as the time to recurrence,^28^ new primary tumor, or death within 5 years of the date of surgery. The all-cause mortality outcome was defined as death of any cause within 1 and 5 years after the date of surgery.

### Statistical Analyses

**Fig. 2:**
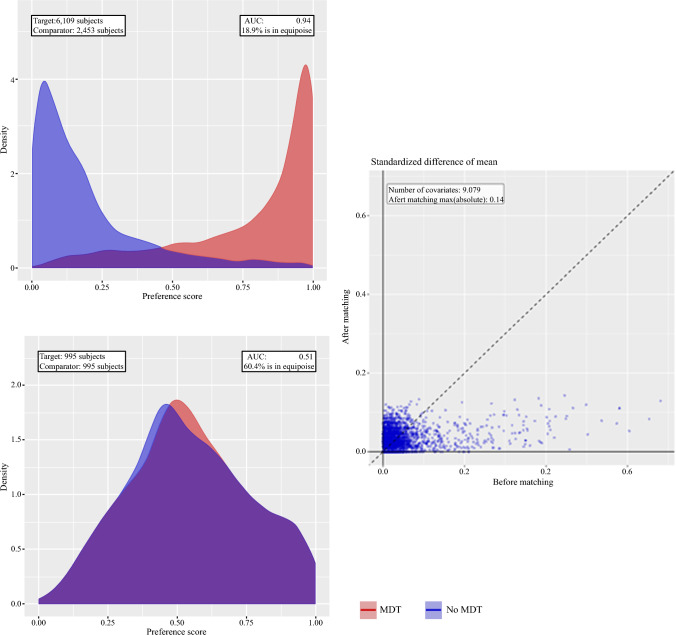
Preference plot before and after propensity score matching for 5-year mortality, and the covariate scatter plot. Remaining preference and covariate scatter plots are shown in electronic supplementary Fig. 1. *AUC* area under the curve

We performed data-driven propensity score matching using large-scale regularized regression to minimize confounding. The ‘MDT’ group was matched with ‘non-MDT’ patients, with matching settings of a 1:1 matching ratio for the closest neighbor. The maximal caliper width was set to 0.2 times the standard deviation of logic.^29,30^ The quality of the propensity score matching was assessed using preference plots before and after matching (Fig. 2), as well as standardized mean differences (SMDs) with a distribution plot as seen in electronic supplementary material (ESM) Fig. 1. The SMD threshold was set according to recommendations in the literature and by the Observational Health Data Sciences and Informatics (OHDSI) community.^20,22,23^

The time-to-event outcomes (i.e., the time to adjuvant therapy component of RIOT, long-term all-cause mortality, and DFS) were assessed using Cox proportional hazard models by calculating the hazard ratio (HR) for the MDT and non-MDT groups. The binary outcomes, namely surgical complications of Clavien–Dindo grade 3b or higher, and the initiation of adjuvant therapy component of RIOT were compared using logistic regression and are presented with odds ratios (ORs) for the outcome (Table 3).

The cohorts were defined using the ATLAS open-source software provided by the OHDSI. For analysis, we used R v.4.0.3 and the PopulationLevelEstimation package,^24^ including CohortMethod v.4.2.2, SelfControlledCaseSeries v.3.2.1, SelfControlledCohort v.1.5.1, and EvidenceSynthesis v.0.2.3. We also used Anaconda3 v. 4.4.0 with Python v. 3.6.10.

### Confounders

The risk of confounding factors was accounted for using propensity score matching. The open-source tool ATLAS utilizes the Cyclops package for R, which includes all patient characteristics, including demographics, conditions, measurements, procedures, and drug or device exposures, in the propensity score matching. Propensity score matching was performed using a data-driven method, whereby the model included all covariates considered relevant in the propensity score matching from each of the four databases.

## Results

The number of patients meeting the inclusion criteria for the target and comparator groups was 26,129, of whom 18,162 were included in the MDT group and 7967 were included in the non-MDT group. For the smaller RIOT cohort, there was a total of 8610 patients, with 6141 in the MDT group and 2469 in the non-MDT-group. After propensity score matching, the larger cohort included 3143 patients in each group, while the smaller RIOT cohort included 995 patients in each group.

Patients were matched based on propensity scores, and the impact of each covariate on the propensity score was measured using SMDs. For selected patient characteristics, SMD values are provided before and after matching (see Tables 1 and 2). Missing data were considered missing at random and there were no imputations for missing data, which were considered not applicable. If there were missing data regarding preoperative MDT, the patient was not included. Due to guidelines from the Danish Health Data Authorities, variables with an incidence below 6 were not shown in the tables due to the risk of patient identification.

**Table 1:** Clinical patient characteristics for all-cause mortality, disease-free survival, and major surgical complications before and after propensity score matching

	MDT discussion cohort		No MDT discussion cohort		SMD
	Before matching [*n* (%)]	After matching [*n* (%)]	Before matching [*n* (%)]	After matching [*n* (%)]	Before/after
Sex					
Female	8160 (45.0)	1628 (51.8)	4121 (51.7)	1645 (52.3)	0.07/−0.01
Male	9969 (55.0)	1515 (48.2)	3853 (48.3)	1498 (47.7)	0.07/0.01
Age, years	Mean: 69.4	Mean: 70.4	Mean: 71	Mean: 70.4	−0.147/0.0016
ASA score					
1	4266 (23.5)	677 (21.5)	1716 (21.5)	711 (22.6)	0.05/−0.03
2	9871 (54.5)	1731 (55.1)	4421 (55.4)	1683 (53.5)	0.02/0.03
3	3630 (20)	661 (21)	1606 (20.1)	648 (20.6)	−0.003/0.01
4	163 (0.9)	30 (1)	58 (0.7)	23 (0.7)	0.02/0.02
5	NA	NA	NA	NA	NA
PS					
0	8994 (49.6)	975 (31)	2285 (28.7)	1083 (34.5)	0.42/−0.07
1	2951 (16.3)	365 (11.6)	907 (11.4)	365 (11.6)	0.14/0
2	860 (4.7)	124 (3.9)	225 (2.8)	115 (3.7)	0.10/0.01
3	186 (1)	21 (0.7)	45 (0.6)	18 (0.6)	0.05/0.01
4	29 (0.2)	7 (0.2)	9 (0.1)	NA	0.01/0.02
CCI					
CCI 0	11,086 (61.2)	1850 (58.9)	4719 (59.2)	1840 (58.5)	0.04/0.006
CCI 1	3264 (18)	575 (18.3)	1478 (18.5)	568 (18.1)	−0.01/0.006
CCI 2	2077 (11.5)	385 (12.2)	981 (12.3)	407 (12.9)	−0.03/−0.02
CCI 3+	1701 (9.4)	333 (10.6)	786 (10)	328 (10.4)	−0.02/0.005
Clinical T staging					
T0	354 (2)	54 (1.7)	74 (0.9)	59 (1.9)	0.06/−0.01
T1	4067 (22.4)	305 (9.7)	405 (5.1)	332 (10.6)	0.37/−0.02
T2	2876 (15.9)	383 (12.2)	627 (7.9)	416 (13.2)	0.17/−0.02
T3	7176 (39.6)	584 (18.6)	973 (12.2)	660 (21)	0.46/−0.05
T4	1747 (9.6)	123 (3.9)	201 (2.5)	147 (4.7)	0.22/−0.03
TX	1688 (9.3)	394 (12.5)	582 (7.3)	398 (12.7)	0.03/−0.003
Clinical N staging					
N0	5621 (31)	712 (22.7)	1147 (14.4)	803 (25.5)	0.28/−0.05
N1	2600 (14.3)	317 (10.1)	527 (6.6)	330 (10.5)	0.18/−0.01
N2	1672 (9.2)	188 (6)	356 (4.4)	229 (7.3)	0.13/−0.04
NX	1958 (10.8)	369 (11.7)	554 (6.9)	385 (12.2)	0.08/−0.01
Clinical M staging					
M0	13,557 (74.8)	2390 (76.1)	6379 (80)	2371 (75.4)	−0.10/0.03
M1	1085 (6)	136 (4.3)	254 (3.2)	141 (4.5)	0.14/−0.001
MX	3486 (19.2)	616 (19.6)	1340 (16.8)	631 (20.1)	0.09/−0.01
Alcohol consumption, units					
0	3774 (20.8)	755 (24)	1892 (23.7)	753 (24)	−0.07/0.001
1–14	10,003 (55.2)	1725 (54.9)	4481 (56.2)	1708 (54.3)	−0.02/0.01
14–21	1439 (7.9)	232 (7.4)	517 (6.5)	219 (7)	0.06/0.02
21+	833 (4.6)	161 (5.1)	418 (5.2)	169 (5.4)	−0.03/−0.01

**Table 2:** Clinical patient characteristics for return to intended oncological therapy before and after propensity score matching

	MDT discussion cohort		No MDT discussion cohort		SMD
	Before matching [*n* (%)]	After matching [*n* (%)]	Before matching [*n* (%)]	After matching [*n* (%)]	Before/after
Sex					
Female Male	2669 (43.5) 3472 (56.5)	457 (45.9) 538 (54.1)	1106 (44.8) 1363 (55.2)	437 (43.9) 558 (56.1)	−0.05/0.02 0.05/−0.02
Age, years	Mean: 69.5	Mean: 71	Mean: 68.7	Mean: 70.5	−0.14/0.01
ASA score					
1 2 3 4 5	1543 (25.1) 3346 (54.5) 1163 (18.9) 49 (0.8) NA	234 (23.5) 536 (53.8) 212 (21.3) 7 (0.7) NA	604 (24.5) 1362 (55.1) 453 (18.4) 15 (0.7) NA	258 (25.9) 528 (53.1) 190 (19.1) 7 (0.7) NA	0.15/-0.06 −0.01/0.02 0.02/0.06 0.03/0 NA
PS					
0 1 2 3 4	3,091 (50.3) 944 (15.4) 270 (4.4) 51 (0.8) 12 (0.2)	312 (31.4) 117 (11.8) 35 (3.5) NA NA	724 (29.3) 272 (11) 69 (2,8) 12 (0.5) NA	54 (35.6) 116 (11.7) 35 (3.5) NA NA	0.42/-0.09 0.13/0.003 0.08/0 0.04/0 -0.002/0.02
CCI					
0 1 2 3+	3947 (63´4.) 1012 (16.5) 640 (10.4) 542 (8.8)	614 (61.7) 177 (17.8) 109 (11.0) 95 (9.5)	1546 (62.6) 406 (16.4) 290 (11.8) 226 (9.2)	616 (61.9) 162 (16.3) 118 (11.9) 99 (9.9)	0.03/−0.004 0.001/0.04 −0.04/−0.03 −0.01/−0.01
Alcohol consumption, units					
0 units 1–14 14–21 21+	1274 (20,7) 3453 (56.2) 482 (7.9) 282 (4,6)	238 (23.9) 578 (58.1) 64 (6.4) 46 (4.6)	552 (22.3) 1435 (58,1) 166 (6.7) 139 (5.6)	225 (22.6) 580 (58.3) 62 (6.7) 56 (1.8)	−0.04/0.03 −0.04/−0.004 −0.04/−0.008 −0.05/−0.05
Clinical staging					
T0 T1 T2 T3 T4 TX N0 N1 N2 NX M0 M1 MX	122 (2) 1389 (22.6) 987 (16.1) 2458 (40) 597 (9.7) 588 (9.6) 2913 (47.4) 1347 (21.9) 864 (14.1) 1017 (16.6) 4583 (74.6) 366 (6) 1192 (19.4)	29 (3) 144 (14.5) 214 (21.5) 314 (31.5) 72 (7.3) 221 (22.3) 457 (46) 186 (18.7) 117 (11.8) 235 (23.6) 754 (75.8) 46 (4.6) 195 (19.6)	63 (2.5) 346 (14) 538 (21.8) 840 (34) 175 (7.1) 506 (20.5) 1093 (44.3) 504 (20.4) 340 (13.8) 532 (21.5) 1968 (79.7) 80 (3.2) 421 (17.1)	30 (3) 162 (16.3) 209 (21) 330 (33.1) 72 (7.3) 192 (19.3) 460 (46.3) 190 (19.1) 130 (13.1) 215 (21.6) 753 (75.6) 42 (4.2) 200 (20.1)	0.06/−0.01 0.37/−0.05 0.17/−0.02 0.45/−0.06 0.22/−0.02 0.02/0.01 0.27/−0.04 0.17/−0.03 0.13/−0.04 0.07/−0.001 −0.10/0.001 0.14/0.02 0.09/−0.02

Propensity score matching was performed for each outcome and yielded three different cohorts (whose characteristics shown in Tables 1 and 2) due to the inclusion time and criteria, as mentioned in the Methods section. This specific propensity score matching was conducted based on 9081–11,758 covariates, depending on the cohort and the outcome. The SMD after propensity score matching was 0.09–0.14, depending on the cohort and the outcome.

*MDT* multidisciplinary team, *ASA* American Society of Anesthesiology, *PS* performance status, *CCI* Charlson comorbidity index, *SMD* standardized mean difference, *T* tumor size stage, *N* number of nodes stage, *M* metastasis stage, *NA* not available

*MDT* multidisciplinary team, *ASA* American Society of Anesthesiology, *PS* performance status, *CCI* Charlson Comorbidity Index, *SMD* standardized mean difference, *T* tumor size stage, *N* number of nodes stage, *M* metastasis stage, *NA* not available

### Short-Term Outcome Results

#### Return to Intended Oncological Therapy

**Fig. 3:**
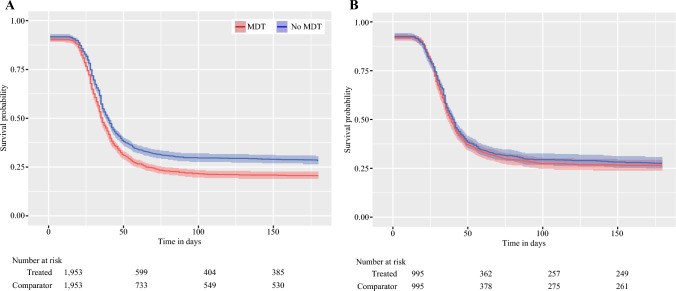
Kaplan–Meier curve for return to intended oncological therapy (**a**) before matching and (**b**) after matching. *MDT* multidisciplinary team

For the entire cohort, the rate of patients returning to intended oncological therapy was 24.1%. For the time-to-event component of RIOT, the HR between the target and comparator groups was 1.246* (95% CI 1.16–1.34) before propensity score matching and 1.04 (95% CI 0.909–1.18) after propensity score matching, with Kaplan–Meier curves presented in Fig. 3a and b, respectively. Additionally, for the binary outcome of the percentage of eligible patients referred for adjuvant therapy, we found that before propensity score matching, 79.3% of patients in the MDT group and 71.9% in the non-MDT group were referred, with an OR of 1.54* (95% CI 1.33–1.78). After propensity score matching, 74.2% and 72.4% of patients in the MDT and non-MDT groups, respectively, were referred, with an OR of 1.02 (95% CI 0.92–1.13). Result times are summarized in Table 3.

**Table 3:** Cohort size of each cohort, median age and follow-up time, and odds or hazard ratios before and after propensity score matching of the target (MDT) and comparator (non-MDT) groups

	No. of patients (each arm)	Median age [target] (IQR)	Median age [comparator] (25th and 75th quantile)	Median follow-up time (25th and 75th quantile)	OR or HR before matching	OR or HR after matching
*Short-term outcomes*						
Major surgical complications	3143	71 (14)	72 (14)		1.03 (95% CI 0.95–1.13)	0.982 (95% CI 0.676–1.43)
RIOT	995	71 (13)	72 (14)		1.246^a^ (95% CI 1.16–1.34)	1.04 (95% CI 0.909–1.18)
*Long-term outcomes*						
1-year mortality	3143	71 (14)	71 (13)	366 (366–366)	0.944 (95% CI 0.821–1.09)	0.952 (95% CI 0.812–1.12)
5-year mortality	3143	71 (14)	71 (13)	1730.5 (962.8–1826)	0.958 (95% CI 0.885–1.03)	0.951 (95% CI 0.868–1.04)
Disease-free survival	3143	71 (13)	71 (13)	1754 (994–1826)	0.952 (95% CI 0.892–1.02)	0.952 (95% CI 0.853–1.06)

*MDT* multidisciplinary team, *IQR* interquartile range, *OR* odds ratio, *HR* hazard ratio, *CI* confidence interval, *RIOT* return to intended oncological therapy

^a^Statistically significant

#### Surgical Complications

For the entire population, the incidence of severe surgical complications was 19.2%. For risk of complications, the OR was 1.03 (95% CI 0.95–1.13) before propensity score matching and 0.982 (95% CI 0.676–1.43) after propensity score matching. These complications were classified as Clavien–Dindo grade 3b or higher. The Kaplan–Meier curves before and after matching are shown in ESM Fig. 2e and f. Result times are summarized in Table 3.

### Long-Term Outcome Results

#### All-Cause Mortality

For 1-year all-cause mortality, the HR was 0.944 (95% CI 0.821–1.09) before propensity score matching and 0.952 (95% CI 0.812–1.12) after matching. For death within 5 years of surgery, the HR was 0.958 (95% CI 0.885–1.03) before propensity score matching and 0.951 (95% CI 0.868–1.04) after matching. Kaplan–Meier curves before and after matching are shown in ESM Fig. 2a and b for 1-year mortality and ESM Fig. 2c and d for 5-year mortality. Results are summarized in Table 3.

#### Disease-Free Survival

For DFS, the HR was 0.952 (95% CI 0.892–1.02) before propensity score matching and 0.952 (95% CI 0.853–1.06) after propensity score matching. Kaplan–Meier curves before and after matching are shown in ESM Fig. 2c and d. The results are summarized in Table 3.

## Discussion

We found no significant differences in major short- and long-term outcomes after CRC surgery for patients discussed at an MDT conference versus patients not discussed at an MDT conference.

It is worth noting that before propensity score matching, a significantly higher HR of 1.246* (95% CI 1.16–1.34) for RIOT was reported in the group discussed at the MDT conference, meaning that among patients with an indication for adjuvant therapy, the event occurred faster and in a greater number of patients when discussed at the MDT (Table 1 and Fig. 3a, b). Similarly, we found that a significantly larger proportion of patients who underwent an MDT conference were referred for adjuvant therapy (79.3% in the MDT group and 71.9% in the non-MDT group; OR 1.54* [95% CI 1.33–1.78]) before propensity score matching. However, similar to the time-to-event analysis, the difference was adjusted by propensity score matching, and 74.2% and 72.4% of patients in the MDT and non-MDT groups, respectively, were referred for adjuvant therapy (OR 1.02 [95% CI 0.92–1.13]) after matching. The largest differences between the two groups were more cases of clinical T3 tumors, rectal cancers, and a performance status of 0 in the MDT group, suggesting systematic differences in the two populations before propensity score matching, which was then amended by matching. For major surgical complications and all long-term outcomes, there was no significant difference between patients in the MDT and non-MDT groups, suggesting that outcomes such as mortality, recurrence, and complications are unrelated to the MDT discussion, or at least by the current way of discussing patients in an MDT conference. The lack of differences between the two groups could be due to the systematic use of clinical guidelines for choice of surgery and indications for neoadjuvant treatment, meaning that with or without a discussion in the MDT conference, the current heavy reliance on guidelines means that patients are offered the treatment suggested by guidelines regardless.

The finding of no significant changes in long- and short-term outcomes after CRC surgery, based on the MDT discussion, correspond with the current literature. Previous Danish studies assessing the effect of MDT conferences on oncological outcomes did not find any significant improvements in the MDT group.^25,26^ However, studies from other countries such as Scotland and Sweden have suggested that MDT conferences have improved DFS. The Scottish study showed 5-year DFS rates of 48.2% in the non-MDT group and 63.1% in the MDT group during the same time frame,^27^ although this study did not explore any of the reasons for this increase.

As the impact of MDT on patient disease courses is still unclear, this may point towards the need to reassess MDT conferences and their structure and information level. In some centers in Denmark, patients with CRC will generally have their first in-person encounter with the colorectal surgeon after the MDT meeting. With treatment decisions primarily made based on referral information, radiologic scans, and histopathological results of varying quality, information on the patients’ wishes is rarely provided.^28^ Thus, the MDT may recommend a certain treatment, while the final decision will always be made between the patient and the treating surgeon in the outpatient clinic. Increasingly, the quality of patient information by, for instance, a visit with a registered nurse before the MDT conference to assess functional capacity and go through the current medication and possibly pre-MDT blood samples, may provide the team with more relevant and necessary information. Of course, this discussion should also include the patients and what they expect and want from their planned treatment. It was not possible to assess whether the incidence of MDT conferences increased after national guidelines encouraged CRC MDT, since this variable was first made available in the DCCG database from 2010.

It has been demonstrated that the introduction of minimally invasive surgery and enhanced recovery after surgery (ERAS) principles has resulted in improved short-term postoperative outcomes.^1,33,34^ However, these interventions mostly focus on the intra- and postoperative period and do not take into consideration how stratification and optimization before surgery can improve the trajectory. Currently, one-third of patients diagnosed with CRC are considered frail and these patients have higher rates of postoperative complications.^35–37^ These patients, if they are not suspected of having metastatic or locally advanced disease, might benefit from local combined endoscopic and laparoscopic surgery (CELS) rather than a full oncological resection to reduce the likelihood of complications,^38^ or might be rescheduled to a later surgery date to leave room for prehabilitation before the procedure.^39^ Some centers have added a geriatrician to the MDT meeting for prehabilitation assessment.^28,40,41^ These personalized interventions could be considered at the MDT conference and might lead to lower rates of postoperative complications. This work has slowly begun by the introduction of the 2023 multimodal prehabilitation guideline from the DCCG database,^42^ including exercise therapy,^36,39,43^ nutritional therapy, and iron infusions,^44–46^ when indicated, however it is rarely possible to decide this before the outpatient meeting with the patient.

A limitation of this study is the relatively small sample of patients in each arm due to exclusion of many patients through propensity score matching, which may lead to selection bias. This is a risk with propensity score matching, especially 1:1, since there are fewer patients in the comparator cohort than in the target cohort. This risk was accepted since the substantial risk of confounding, if not using propensity score matching, on these generally different populations was considered to be more harmful to the validity and usability of the study. Additionally, as this was a retrospective observational study, there is a risk of missing data and information bias. As a limitation, it should be mentioned that the CRC MDT conference in Denmark included both colon and rectum surgeons and oncologists, leading to a less sharper distinction between the colon cancer and rectum cancer MDT, which makes it difficult to abruptly split the two and assess the efficiency of MDT in colon cancer and rectum cancer. Another limitation of this study is that as previously stated, multiple different MDT conferences, including postoperative, metastatic, and recurrence conferences, exist; however, the current data sources scarcely register these MDT modalities and therefore this study could only focus on the impact of preoperative MDT conferences. Using propensity score matching yields more comparable case and control groups, mimicking the scenario of a randomized controlled trial (RCT), but is of course not as controlled as a true RCT. To have ideal propensity score matching, the literature suggests an SMD below 0.1.^22,23^ For the four outcomes, i.e. 1- and 5-year mortality, DFS, and major surgical complications, the SMD was 0.09 (ESM Fig. 1), which fulfills this criteria, however for the RIOT cohorts, the SMD was 0.14, which despite being close is not quite below 0.1, meaning that although matched on propensity score, there are still some differences in the two groups. It is however important to note that the cohorts were propensity score matched on 9079 covariates for RIOT and 11,541 covariates for the remaining covariates, meaning that although the SMD is slightly above 0.1, the groups were very similar across thousands of covariates. Additionally, although the time frame of ≤60 days before the date of surgery for the MDT conference to take place was set to accommodate the possibility of neoadjuvant therapy before rectal cancer surgery, it was out of the scope of this study to explicitly investigate the impact of neoadjuvant therapy. Lastly, another limitation was that for MX patients, the data did not clearly clarify what the suspected metastases were and how they were considered relevant for curative therapy. The likely reason is the incidence of unspecific lung nodules, which were followed-up with computer-assisted tomography scans of the thorax 3 months after surgery.

The strengths of this study include the highly granular propensity score matching, lowering the risk of confounding. Second, we used the OMOP-CDM and its applicable analysis tools in ATLAS, which are well-described and validated internationally. The use of the unified CDM for the DCCG database, DNPR, RLRR, and NPR provides a data foundation with information regarding multiple facets of a CRC trajectory with a high level of detail.

## Conclusions

Since the Danish Multidisciplinary Cancer Group introduced recommendations that all patients with malignancy should be discussed in MDT conferences, we found that more than two-thirds of patients undergoing CRC surgery were also discussed in an MDT conference. We found no significant differences in short- and long-term oncological outcomes after CRC surgery in patients discussed at MDT conferences compared with those who were not. Further research into newer tools for personalization of care is needed to assess if differentiated care conferences can improve patient outcomes.

## Supplementary Information

Below is the link to the electronic supplementary material.

Supplementary file 1 (DOCX 198 kb)

## Data Availability

The data sources included in the OMOP-CDM were the DCCG database provided by the Danish Clinical Quality Program, and the DNPR, Danish Laboratory Research Database, and Danish Medicinal Product Statistics Database, all provided by the Danish Health Data Authority.
