# Supplementary material for: The Association Between Conducting a Multidisciplinary Team Conference and Short- and Long-Term Outcomes After Colorectal Cancer Surgery: A National Register Study
Source: Ann Surg Oncol. 2025 Sep 30;32(13):9491–501. doi: 10.1245/s10434-025-18353-y (PMC12589314; doi:10.1245/s10434-025-18353-y)
Supplement: Supplementary file 1 — (DOCX 198 kb) [file 10434_2025_18353_MOESM1_ESM.docx]

**SUPPLEMENTARY FIG. 1** Covariate balance plot for the cohort used to estimate the hazard of 1-year mortality, 5-year mortality, 5-year disease-free survival, and 30-day major surgical complications


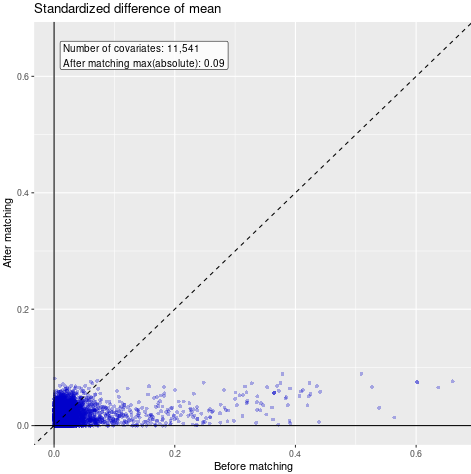


**SUPPLEMENTARY FIG. 2** Kaplan–Meier curves for (**a**) 1-year mortality before matching; (**b**) 1-year mortality after matching; (**c**) 5-year mortality before matching; (**d**) 5-year mortality after matching; (**e**) 5-year disease-free survival before matching; and (**f**) 5-year disease-free survival after matching

*
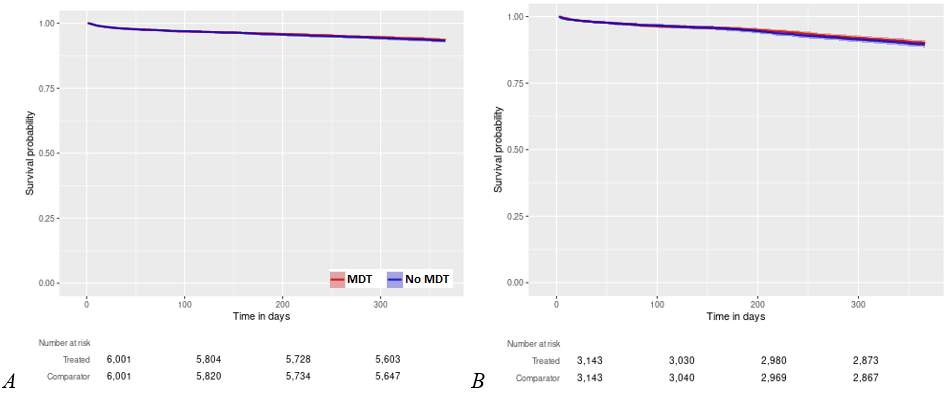
*

*
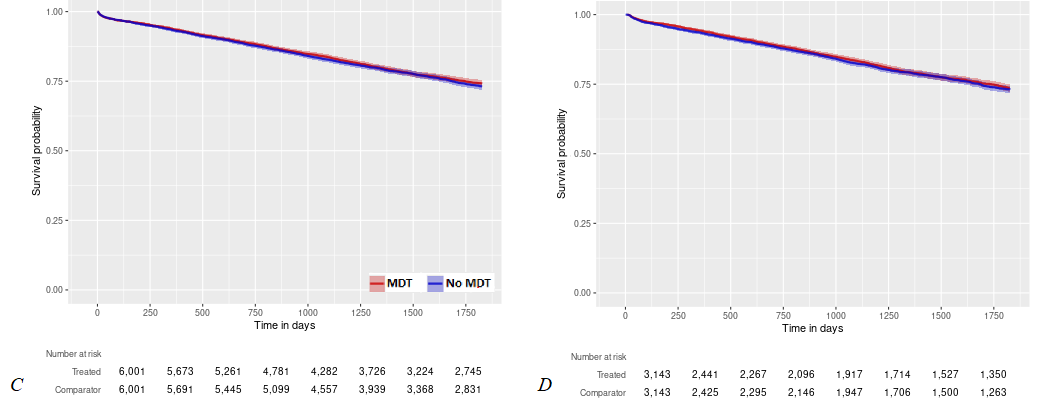
*

*
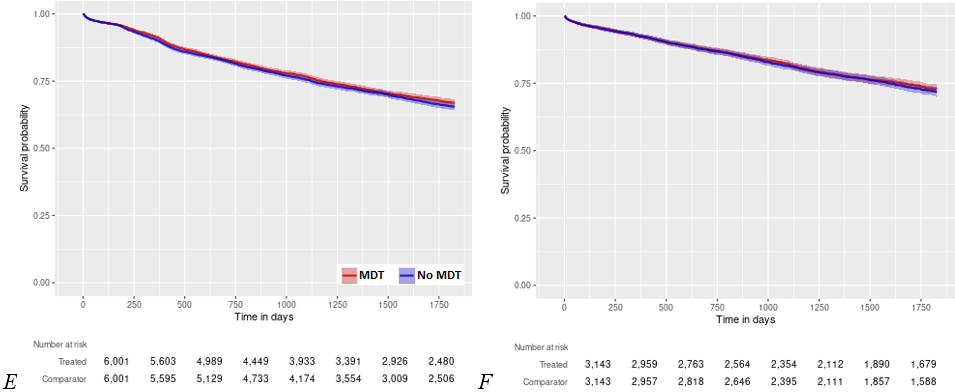
*
